# Genetics of exceptional longevity: possible role of GM allotypes

**DOI:** 10.1186/s12979-018-0133-8

**Published:** 2018-11-06

**Authors:** Calogero Caruso, Janardan P. Pandey, Annibale A. Puca

**Affiliations:** 1 Department of Pathobiology and Medical Biotechnologies, Section of General Pathology, University of Palermo, Corso Tukory 211, 90134 Palermo, Italy; 2 Department of Microbiology and Immunology, Medical University of South Carolina, Charleston, SC 29425 USA; 3 Department of Medicine and Surgery, University of Salerno, Baronissi, Italy; 4 IRCCS MultiMedica, Milan, Italy

**Keywords:** Case control studies, GM allotypes, GWAS, Immune response, Longevity

## Background

Many variables contribute to the way we age and the consequent possible achievement of extreme ages. Among these, we can include cultural, anthropological and socio-economic status as well as sex and gender (women live longer than men). Also, ethnic differences (explained by discrepancies in healthcare, environmental and economic status, genetics as well as life occupation) exist in relation to ageing/longevity. In any case, the concrete possibility to manifest a longevity phenotype is strictly related to the stochastic interactions due to accidental events, with environmental and genetic factors having a role in ageing and longevity processes. The genetic component is progressively a major determinant as we evaluate extreme ages to be reached. Indeed, the possibility to inherit longevity increases with age: for long living individuals (LLIs), which are people that belong to the top 5th percentile of life-spans (i.e. 95 years in males and 98 years in female), it reaches up to 33% for women and 48% for men [1–3] (https://www.ssa.gov/OACT/STATS/, under “life table”).

Genetics of exceptional longevity has been developed with different approaches depending on the available technologies and on the costs for a single analysis. Although the reduced costs have allowed more comprehensive studies, more is not always better. As an example, given the number of samples available for a given genetic analysis, the power of the study is reduced progressively as we add more hypothesises to test, to the point that the study becomes underpowered in genome-wide association studies (GWAS) [4].

In other words, there is no simple equation, such as more hypotheses = more results. Indeed, APOE ε4 allele was associated with exceptional longevity in 1994 through a candidate gene approach using a small sample size [5]. Since then, many replication attempts were successful, despite the fact that there is a strong gradient in terms of ε4 allele frequency among Northern and Southern European studies (lower in the South) [6]. APOE was so strongly associated that it came up in most GWAS for exceptional longevity, surviving the Bonferroni’s correction of the threshold of significance adopted for GWAS (*p* < 5 × 10^− 8^). This is also true for the FOXO3A locus, while for others replication attempts were not consistent, possibly due to the multiple testing that reduced the threshold of significance [7, 8]. Thus, in the era of candidate gene studies in exceptional longevity, the limitation was not underpowered studies, but the lack of correction for genetic admixture. Indeed, stratification, which is the bias that brings to an enrichment of a specific ethnicity in one of the two arms (cases and controls) of the study, is the main cause of false positive results [4].

In an attempt to reconcile the results of different studies, statistics that included different studies (meta-analysis) was developed. Still, the non-homogeneous criteria adopted to select the two arms of a case-control study could result in conflicting results. Among the criteria that vary among the studies, it is important to mention the ages of LLIs and the young controls and gender distribution, parameters that could influence associations of genetic variants that intervene at extreme ages and with a gender effect [9].

Recently, many studies have been accomplished using chip arrays that interrogated hundreds of thousands of single nucleotide polymorphisms (SNPs,) followed by the imputation of the missing SNPs allowing a comprehensive analysis of the entire genome, with some exceptions where polymorphisms were not well represented at the chip level. Furthermore, the Bonferroni’s correction is a strong killer for genetic risk factors with small effects unless the number of individuals tested is in the tens of thousands. The LLIs are healthy individuals that are not hospitalized and are recruited by home visiting, making it difficult to reach large numbers [4].

## Discussion

Candidate gene studies involving the genomic regions that are not well represented in the SNP arrays are therefore welcome for the discovery of new potential associations with exceptional longevity. It is useful to adopt populations that have already been used for GWAS so as to exclude stratification effects. This is indeed the case for the study presented by Puca et al., [10] which analysed the role of genetic markers of γ chains (GM allotypes), i.e. the hereditary antigenic determinants expressed on immunoglobulin G polypeptide chains, in the attainment of longevity. In this study, the DNA samples from 95 LLIs (mean age 96.7) and 96 young controls (mean age 31.9) from South Italy were typed for GM3/17 and GM23+/− alleles, showing that GM3 allotype is significantly overrepresented in both male and female LLIs. The rs1071803 SNP that codes for the GM 3/17 (arginine/lysine) allotypes is not represented in the commonly employed genotyping platforms. It can be imputed, but the quality of imputation is poor.

Literature data show that human longevity may be correlated with optimal functioning of the immune system, so suggesting that genetic determinants of longevity also resides in those polymorphisms for the immune system genes that regulate immune responses, such as human leukocyte antigen (HLA) [11, 12]. Accordingly, several studies have examined the role of HLA antigens in longevity [11, 13]. It has been known since at least 1971 [14] that GM allotypes contribute to the interindividual differences in the magnitude of immune responsiveness, so it is not surprising that GM allotypes are seemingly associated with longevity, it is instead surprising that until now no study was performed on GM allotypes and longevity.

As first suggested by J.B.S. Haldane [15], major infectious diseases have been the principal selective forces in shaping our evolutionary history. GM allotypes have been shown to be associated with immune responsiveness to several major infectious pathogens and with survival from epidemics [16]. One mechanism for how GM determinants could contribute to the outcome of infection with various agents may be through allotype-mediated antibody responses against pathogens, resulting in differential immunity to infectious diseases. Thus, GM allotypes could participate as recognition structures for the pathogenic epitopes on B cell membranes. Additionally, and contrary to the prevalent belief in immunology, these constant-region determinants could directly influence antibody specificity by causing conformational changes in the antigen-binding site in the immunoglobulin variable region. They could also influence the expression of idiotypes involved in immunity to the pathogens. Contribution of both variable and constant regions in the formation of idiotypic determinants was documented many years ago [17].

## Conclusion

Human population is very heterogeneous because of the different genetic background and different environmental stimuli, so it has not yet been possible to identify a clear signature of longevity with the exception of APOE and FOXOA. The study of Puca et al., [10] was performed in a very homogeneous population from South Italy, so the observed association of GM with longevity should not depend on population stratification. However, further studies are necessary to confirm this association. GM17/17 (the alternative allele of GM3) has been shown to be associated with the risk of developing HCMV symptomatic infection [18]. Considering the role of HCMV in immunosenescence [19, 20], future studies might evaluate antibodies titers directed versus HCMV in LLIs and young controls, according to GM3 allotype.

## Abbreviations

APOE: apolipoprotein E
GM: genetic markers of γ chains
GWAS: genome-wide association studies
HCMV: human cytomegalovirus
HLA: human leukocyte antigen
LLI: Long-living individual
SNP: single nucleotide polymorphism
